# BCL-1 Gene Rearrangements in Iranian Non-Hodgkin Lymphoma Patients

**DOI:** 10.5539/gjhs.v8n8p108

**Published:** 2015-12-17

**Authors:** Manoush Tohidirad, Mehrdad Asghari Estiar, Azim Rezamand, Saeid Ghorbian, Sasan Andalib, Issa Jahanzad, Tayyeb Bahrami, Ebrahim Sakhinia

**Affiliations:** 1 Department of Basic Science, East Azarbayjan Science and Research Branch, Islamic Azad University, Tabriz, Iran; 2 Department of Basic Science, Tabriz Branch, Islamic Azad University, Tabriz, Iran; 3 Students’ Scientific Research Center, Department of Medical Genetics, School of Medicine, Tehran University of Medical Sciences, Tehran, Iran; 4 Department of Pediatrics, Children Hospital, Tabriz University of Medical Sciences, Tabriz, Iran; 5 Department of Molecular Biology, Ahar Branch, Islamic Azad University, Ahar, Iran; 6 Neurosciences Research Center, Imam Reza Hospital, Tabriz University of Medical Sciences, Tabriz, Iran; 7 Department of Pathology, Imam Khomeini Hospital Complex, Tehran University of Medical Sciences, Tehran, Iran; 8 Genetic Research Center, University of Social Welfare Science & Rehabilitation Sciences, Tehran, Iran; 9 Biotechnology Research Center, Tabriz University of Medical Sciences, Tabriz, Iran

**Keywords:** BCL-1, non-hodgkin lymphoma, FFPE, translocation, BIOMED-2 protocol

## Abstract

In the present study, our aim was to assess the incidence of BCL-1 gene rearrangements in formalin-fixed paraffin embedded (FFPE) tissue in patients with non-Hodgkin lymphomas (NHL). The BIOMED-2 protocol was applied to assess the BCL-1 gene rearrangements in NHL patients. PCR amplification was carried out on FFPE in 100 patients with B-cell lymphoma including 89 cases with diffused large B-cell lymphoma (DLBCL) (15 cases under 18 years old) and 11 cases with mantle cell lymphoma (MCL). Out of the 100 patients, 19 cases (19%) were identified to have concurrent translocation involving BCL-1. The significant association was seen between BCL-1 gene rearrangements and the lymphomas in patients older than 55 years (P<0.05). Out of 100 cases, 80 cases were positive and 20 cases were negative regarding CD20. No significant association was found between DLBCL lymphoma in patients under 18 years old and BCL-1 gene rearrangements (P>0.05). In addition, the positive and negative expressions of LCA/CD45 marker were 76% (76/100) and 26% (26/100), respectively. Our findings revealed that BCL-1 gene rearrangement assays using BIOMED-2 protocol can be considered as a valuable approach in detection of the lymphomas.

## 1. Introduction

Human lymphomas, aggressive malignant disorders, are categorized into the Hodgkin and non-Hodgkin lymphoma (NHL) according to World Health Organization (WHO) (Jaffe, 2001; Swerdllow et al., 2008). NHL includes several histologically and biologically different lymphoid malignancies, which currently are less divulged with possibly distinct etiologies (Chiu & Hou, 2015). It is estimated that there were approximately 70800 new cases of NHL leading to 18990 deaths, making up for an about 4% of new cancer diagnoses and 3% of cancer deaths, in the USA (Siegel et al., 2014). Roughly 85% of NHL cases show a B-cell origin. Most common subcategories of NHL include aggressive diffuse large B-cell lymphoma (DLBCL), indolent follicular lymphoma (FL), and mantle cell lymphoma (MCL) (Shankland et al., 2012; Swerdlow et al., 2008). The balanced translocations (Chaganti et al., 2000) in NHL show distinct disease entities (Huang et al., 2002; Barrans et al., 2002). Therefore, assessment of such lesions should include a more precise diagnosis and a risk-based treatment.

Cytogenetic analyses demonstrated that there is a close association between MCL and t (11; 14) (q13; q32) (Leroux et al., 1991; Vandenberghe et al., 1992). The translocation juxtaposes sequence of Ig heavy chain gene (IGH) with the BCL*-*1 locus, which results in the CCND1 gene up-regulation and, in turn, cyclin D1 overexpression (Rimokh et al., 1993; de Boer et al., 1995; de Boer et al., 1995); although concordancy was not shown between overexpression of cyclin D1 and t (11; 14) (q13; q32) in MCL cases (Leroux et al., 1991; Vandenberghe et al., 1992). Such discrepancy can be due to a low mitotic index of malignant cells and the metaphase spreads’ poor morphology. Additionally, cytogenetic assessment is a time-consuming method, especially, in the assessment of lymphoma samples (Li et al., 1999). Other molecular methods show their own restriction scattering 11q13 breakpoints. Morphological features along with immunopathological assessments are now routinely, the principle of diagnosis in neoplastic lymphoma, whereas, the availability of BIOMED-2 protocol further enhances our ability to diagnose and classify lymphoid malignancies.

Currently, the BIOMED-2 multiplex PCR methods, based on clonality assays, are being widely applied in the diagnosis of suspected lymphoproliferative disorders (LPD). Thus, in this study, we applied the BIOMED-2 PCR protocol to evaluate the rate of CCND1-IGH fusion in NHL patients in order to improve the NHL detection.

## 2. Materials and Methods

Samples including 100 formalin-fixed paraffin-embedded (FFPE) blocks were obtained from the archives of the Department of Pathology at Cancer Institute of Imam Khomeini Hospital, Tehran, Iran. And, the analyses were carried out at Tabriz University of Medical Sciences. The pathological board re-assessed all samples and confirmed the diagnosis based on WHO classification criteria. Informed consent had been obtained from each participant and the utilized protocol was approved by the ethic committee, which was in compliance with the Helsinki declaration. As controls, tonsil tissue and IVS-0010 (5%) control clonal DNA (InvivoScribe; catalog No. 4-088-0590) used as positive sample. Three or four 5-micron sections of the samples were obtained from each FFPE tissue, transferred into sterile containers and stored at room temperature. Thereafter, DNA was extracted as described by manufacturer’s protocol (Quick Extract kit, UK). Briefly, all the samples incubated at 98 °C for 2 min followed by 56 °C for 1 h in 1.5 ml sterile tubes containing 100 μl of extraction kit solution. Tubes were shaken every 30 min. The samples were then centrifuged at 8870 g for 10 min. Finally, collected samples were stored at -20 °C. In order to evaluate the quality and quantity of the extracted DNA, all samples were quantified by UV spectrophotometry at 260/280 nm (using the NanoDrop TM ND-1,000, NanoDrop Technology, Wilmington, DE, USA). The average concentration of all DNA and OD260/280 ratio were 150 ng/μl and 1.85, respectively.

For the detection of t (11; 14) translocations, based on BIOMED-2 protocol, a pair of primers (Table 1) was used for PCR amplification.

**Table 1:** Primers used for the PCR amplification

Name	Sequence
Bcl1/MTC	5’ GGATAAAGGCGAGGAGCATAA 3’
JH consensus	3’ CCAGTGGCAGAGGAGTCCATTC 5’

For determination of breakpoint at cluster region, PCR were carried out on the extracted DNA of the tested samples, negative and positive controls. The PCR reactions were in a final volume of 25μl containing 150 ng genomic DNA, 1 pmol of each primer, and 14 μlred master mix (Ampliqon, Denmark). Amplification program was initiated with pre-incubation at 95°C for 10 min, followed by 35 cycles of 30 s at 95°C, 50 s at 60°C, and 90 s at 72°C. After the final cycle, a post-extension step was performed for 10 min at 72°C. Subsequently, PCR products were directly loaded on the agarose gel (1.5%) and stained with ethidium bromide.

## 3. Results

PCR amplification assay was performed with a pair of primers to rapidly identifyCCND1-IGH fusion genes stemming from breakpoints in MTC. If additional bands except the control fragment were amplified, PCR assays would be carried out. This reaction detected MTC breakpoints. Thus, amplification only identified a CCND1-IGH fusion. A total of 100 lymph node cases that consisted of 89 DLBCL cases including 15 cases less than 18 years old and 11 cases of MCL were identified. Patients ages ranged between 13 to 91 years old at the time of diagnosis (mean 50.95 years). The patients included 63% males and 37% females.

Out of 100 cases, 19 cases (19%) were observed to carry a concurrent translocation involving BCL-1 based on PCR analysis using BIOMED-2 protocol. There was a significant association between BCL-1 gene rearrangements and the lymphomas in the patients older than 55 years (P<0.05). Gender was not significantly associated with BCL-1 gene rearrangements (P>0.05). There was not significant association between DLBCL lymphoma patients under 18 years old and BCL-1 gene rearrangement (P>0.05).

While investigating the two markers, 80% (80/100) of the cases were positive and 20% (20/100) of cases were negative for CD20. In addition, the positive and negative expressions of LCA/CD45 marker were 76% (76/100) and 26% (26/100), respectively.

**Table 2:** Immunohistochemical and clinicopathological features of 19 patients with BCL-1 gene rearrangements

ID	Age at Diagnosis	Sex	Histological Type	CD20	LCA/CD45
**M3-1**	61	F	DLBCL	+	-
**M3-2**	73	F	MCL	+	+
**M3-3**	59	M	MCL	+	+
**M3-5**	62	M	DLBCL	+	-
**M3-6**	74	F	DLBCL	-	+
**M3-10**	57	F	MCL	+	+
**M3-11**	70	M	MCL	-	+
**M3-12**	13	M	DLBCL	+	+
**M3-13**	70	F	DLBCL	_	+
**M3-17**	45	M	MCL	+	-
**M3-24**	75	F	DLBCL	_	-
**M3-26**	50	M	MCL	_	+
**M3-34**	70	M	MCL	+	-
**M3-39**	71	M	DLBCL	+	+
**M3-40**	67	M	DLBCL	+	-
**M3-66**	62	F	DLBCL	-	+
**M3-68**	59	F	DLBCL	-	+
**M3-70**	22	F	DLBCL	+	+
**M3-71**	48	M	DLBCL	-	-

## 4. Discussion

There is an increasing amount of literature demonstrating that balanced translocations are available in a large and heterogeneous number in NHL. Lymphoid cells experience surface antigen receptor gene rearrangements over immune cell system differentiation. Nevertheless, tumor cells showed identical immunoglobulin gene rearrangements patterns based on the origin of B, T, and NK cells in lymphoid malignancies (Van Dongen et al., 2003; Evans et al., 2006; Langerak et al., 2012; Isola et al., 1994; Langerak et al., 2012; Shan et al., 2013). Mantle cell lymphoma (MCL), which refers to a different type of B-cell lymphoma, is t(11;14) translocation and cyclin D1 overexpression (Caballero et al., 2013). Furthermore, various translocations were shown in smaller fractions of individuals with lymphoma involving C-MYC/IGH (t(8;14)(q24;q32)), IGK/C-MYC (t(2;8)(p12;q24)), and C-MYC/IGL (t(8;22)(q24;q11)) in Burkitt lymphoma, API-2/MLT (t(11;18)(q21;q21)) and BCL-10/IGH (t(1;14)(p22;q32)) in MALT lymphoma, BCL-6/VARIOUS (t(3;Var)(q27;Var)) and IGH/BCL-8 (t(14;15)(q32;q11-13)) in DLCL, ALK/NPM-1 (t(2;5)(p23;q35)) in anaplastic large cell lymphoma, and PAX-5/IGH(t(9;14)(p13;q32)) in LL (Medeiros & Carr, 1999).

FISH and PCR are molecular cytogenetic techniques for assessment of these translocations particularly t(11;14)(q13;q32) with formation of the fusion oncogene IgH/BCL1 in MCL lymphomas (Chu et al., 2011). In spite of the fact that PCR is the most sensitive and specific molecular technique to determine genetic aberrations through chromosome rearrangements, FFPE tissue samples are known to be a reliable source of DNA for molecular assessment (Tajana et al., 2010).

We previously reported that the BIOMED-2 protocol could be considered reliable for clonality detection, especially in IGH analysis on FFPE in lymphoid malignancies (Moharrami et al., 2014). We also found that the complete IGH and incomplete IGH D-J clonality gene rearrangement assays using BIOMED-2 protocols may be valuable for detection of clonal gene rearrangements, especially in HL cases (Ghorbian et al., 2015). IGH and IGK rearrangement based upon BIOMED-2 protocol enhanced clonality detection rate (up to 25% of cases) in lymphoma malignancies (Tapia et al., 2012; Ghorbian et al., 2014). We described a molecular method for effective identification of CCND1-IGH fusion in DLBCL (Ghorbian et al., 2014).

In this study, 100 paraffin embedded lymph node specimens were analyzed for the t(11;14) Bcl-1 gene rearrangement using PCR to detect the t(11;14) translocation by one pair of primers specific for the MTC breakpoint region from the BIOMED-2 protocol. Our results showed that 19 out of 100 cases were t(11;14) positive. It was reported that the positive rates of t(11;14) translocation analyzed by general and semi-nested PCR in MCL were 25.81% and 35.48%, respectively(Chu et al., 2011). In the present study, patients older than age of 55 years appeared to be more likely to carry the t(11;14)(q13;q32). The increase in the incidence of the t(11;14) translocation with age in adults has been confirmed elsewhere (Bijal et al., 2012). In conclusion, our findings show an accurate detection of t(11;14) translocation by using PCR with one series of breakpoint (BIOMED2 protocol), which is useful to assess an appropriate initial therapy and the neoplasms’ management. Hence, further investigations with new set of molecular categories are required to assess possible correlations with clinical data and offer a better understanding of the biology, diagnostic and treatment of this neoplasia.

## References

[ref1] Barrans S. L, O’Connor S. J, Evans P. A, Davies F. E, Owen R. G, Jack A. S (2002). Rearrangement of the BCL6 locus at 3q27 is an independent poor prognostic factor in nodal diffuse large B-cell lymphoma. Br J Haematol.

[ref2] Bijal S, Martin P, Eduardo M. S (2012). Mantle Cell Lymphoma:A Clinically Heterogeneous Disease in Need of Tailored Approaches. Cancer control:Journal of the Moffitt Cancer Center.

[ref3] Caballero D, Campo E, López-Guillermo A, Martín A, Arranz-Sáez R, Giné E, …Orfao A (2013). Clinical practice guidelines for diagnosis, treatment, and follow-up of patients with mantle cell lymphoma. Recommendations from the GEL/TAMO Spanish Cooperative Group. Ann Hematol.

[ref4] Chaganti R. S, Nanjangud G, Schmidt H, Teruya-Feldstein J (2000). Recurring chromosomal abnormalities in non-Hodgkin’s lymphoma:Biologic and clinical significance. Semin Hematol.

[ref5] Chiu B. C, Hou N (2015). Epidemiology and etiology of non-hodgkin lymphoma. Cancer Treat Res.

[ref6] Chu H, Han X, Jiang H, Li F, Li H, Zhao T (2011). Nuclei micro-array FISH, a desirable alternative for MCL diagnosis. Ann Hematol.

[ref7] de Boer C. J, Schuuring E, Dreef E, Peters G, Bartek J, Kluin P. M, van Krieken J. H. J. M (1995). Cyclin D1 protein analysis in the diagnosis of mantle cell lymphoma. Blood.

[ref8] de Boer C. J, van Krieken J. H. J. M, Kluin-Nelemans H. C, Kluin P. M, Schuuring E (1995). Cyclin D1 messenger RNA overexpression as a marker for mantle cell lymphoma. Oncogene.

[ref9] Evans P, Pott C, Groenen P, Salles G, Davi F, Berger F, Cabecadas J (2006). Significantly improved PCR-based clonality testing in B-cell malignancies by use of multiple immunoglobulin gene targets. Report of the BIOMED-2 Concerted ActionBHM4-CT98-3936. Leukemia.

[ref10] Ghorbian S, Jahanzad I, Estiar M. A, Eivazi Ziae J, Asvadi-Kermani I, Andalib S, Sakhinia E (2015). Molecular Analysis of IGH and Incomplete IGH D-J Clonality Gene Rearrangements in Hodgkin Lymphoma Malignancies. Clin Lab.

[ref11] Ghorbian S, Jahanzad I, Javadi G. R, Sakhinia E (2014). Evaluation Diagnostic Usefulness of Immunoglobulin Light Chains (Igκ, Igλ) and Incomplete IGH D-J Clonal Gene Rearrangements in Patients with B Cell Non-Hodgkin Lymphomas Using BIOMED-2 Protocol. Clin & Trans Oncol.

[ref12] Huang J. Z, Sanger W. G, Greiner T. C, Staudt L. M, Weisenburger D. D, Pickering D. L, Chan W. C (2002). The t(14;18) defines a unique subset of diffuse large B-cell lymphoma with a germinal center B-cell gene expression profile. Blood.

[ref13] Isola J, DeVries S, Chu L, Ghazvini S, Waldman F (1994). Analysis of changes in DNA sequence copy number by comparative genomic hybridization in archival paraffin-embedded tumor samples. Am J Pathol.

[ref14] Jaffe E. S (2001). Pathology and genetics of tumours of haematopoietic and lymphoid tissues. Iarc.

[ref15] Langerak A. W, van Dongen J. J (2012). Multiple clonal Ig/TCR products:implications for interpretation of clonality findings. J Hematopathology.

[ref16] Langerak A, Groenen P, Bru¨ggemann M, Beldjord K, Bellan C, Bonello L, Van Dongen J. J. M (2012). EuroClonality/BIOMED-2 guidelines for interpretation and reporting of Ig/TCRclonality testing in suspected lymphoproliferations. Leukemia.

[ref17] Leroux D, Le Marc’hadour F, Gressin R, Jacob M. C, Keddari E, Monteil M, Sotto J. J (1991). Non-Hodgkin’s lymphomas with t(11;14)(q13;q32):A subset of mantle/intermediate lymphocytic lymphoma?. Br J Haematol.

[ref18] Li J. Y, Gaillard F, Moreau A, Harousseau J. L, Laboisse C, Milpied N, Avet-Loiseau H (1999). Detection of Translocation t(11;14)(q13;q32) in Mantle Cell Lymphoma by Fluorescence in Situ Hybridization. Am J Pathol.

[ref19] Medeiros L. J, Carr J (1999). Overview of the role of molecular methods in the diagnosis of malignant lymphomas. Arch Pathol Lab Med.

[ref20] Moharrami G, Ghorbian S, Seifi M, Estiar M. A, Fakhrjoo A, Sakhinia M, Sakhinia E (2014). Detection of Immunoglobulin IGH Gene Rearrangements on formalin-Fixed, Paraffin Embedded Tissue in Lymphoid Malignancies. Cell Mol Biol.

[ref21] Rimokh R, Berger F, Delsol G, Chanin C, Bertheas M. F, French M, Magaud J. P (1993). Rearrangement and overexpression ofthe bcl-1/PRAD-1 gene in intermediate lymphocytic lymphomas and int(11q13)-bearing leukemias. Blood.

[ref22] Shan G. D, Hu F. L, Yang M, Chen H. T, Chen W. G, Wang Y. G, Xu G. Q (2013). Clonal immunoglobulin heavy chain and T-cell receptor c gene rearrangements in primary gastric lymphoma. World J Gastroenterol WJG.

[ref23] Shankland K. R, Armitage J. O, Hancock B. W (2012). Non-Hodgkin lymphoma. Lancet.

[ref24] Siegel R, Ma J, Zou Z, Jemal A (2014). Cancer statistics, 2014. CA Cancer J Clin.

[ref25] Swerdllow S, Campo E, Harris N. L (2008). WHO classification of tumours of haematopoietic and lymphoid tissues.

[ref26] Tajana L. A, Ajdukovi R, Pej V, Ku R (2010). Detection of t(14;18) by PCR of IgH/BCL2 Fusion Gene in Follicular Lymphoma from Archived Cytological Smears. Coll Antropol.

[ref27] Tapia G, Sanz C, Mate J. L, Muñoz-Mármol A. M, Ariza A (2012). Improved clonality detection in Hodgkin lymphoma using the BIOMED-2-based heavy and kappa chain assay:A paraffin-embedded tissue study. Histopathology.

[ref28] Van Dongen J, Langerak A, Bru¨ggemann M, Evans P, Hummel M, Lavender F, Macintyre E. A (2003). Design and standardization of PCR primers and protocols for detection of clonal immunoglobulin and T-cell receptor gene recombinations in suspect lymphoproliferations:report of the BIOMED-2 Concerted Action BMH4-CT98-3936. Leukemia.

[ref29] Vandenberghe E, de Wolf-Peeters C, Wlodarska I, Stul M, Louwagie A, Verhoef G, van den Berghe H (1992). Chromosome 11q rearrangements in B non-Hodgkin’s lymphoma. Br J Haematol.

